# Humic Acid Modulates Photosynthetic Responses to PEG-Induced Drought in *Ocimum basilicum* L.

**DOI:** 10.3390/plants15101491

**Published:** 2026-05-13

**Authors:** Martin A. Stefanov, Georgi D. Rashkov, Preslava B. Borisova, Anelia G. Dobrikova, Emilia L. Apostolova

**Affiliations:** Institute of Biophysics and Biomedical Engineering, Bulgarian Academy of Sciences, Acad. G. Bonchev Str., Bl. 21, 1113 Sofia, Bulgaria; martin_12.1989@abv.bg (M.A.S.); megajorko@abv.bg (G.D.R.); aneli@bio21.bas.bg (A.G.D.)

**Keywords:** antioxidant enzymes, basil, chlorophyll fluorescence, drought tolerance, non-enzymatic antioxidants, photosynthetic efficiency

## Abstract

Drought is a major environmental constraint that disrupts photosynthetic processes. This study investigated the effects of foliar-applied commercial humic acid (HA) at different concentrations (1, 3 and 5 mg/mL) on the photosynthetic apparatus of sweet basil (*Ocimum basilicum* L. Italiano classico) under PEG-induced stress. The responses of the photosynthetic machinery were evaluated using chlorophyll a fluorescence analyses (JIP-test and PAM), leaf pigment composition, and assessments of membrane integrity. Drought stress caused pronounced alterations on both the donor and acceptor sides of photosystem II (PSII), including impaired Q_A_^−^ reoxidation, reduced open PSII reaction centers (qP), diminished electron transport (ETo/RC, REo/RC), and substantial declines in performance indices (PI_ABS_, PItotal). Energy dissipation increased (DI_0_/RC), with regulated energy losses (Φ_NPQ_) rising more strongly than non-regulated losses (Φ_NO_). Drought also elevated oxidative stress markers (MDA and H_2_O_2_), leading to enhanced membrane injury. Among the tested concentrations, 5 mg/mL HA provided the most effective protection against drought stress. This treatment mitigated PEG-induced damage on both PSII donor and acceptor sides and increased the proportion of open reaction centers (qP). Improved PSII photochemistry corresponded with more efficient Q_A_^−^ reoxidation, facilitated its interaction with plastoquinone, and caused the overall stabilization of photosynthetic functions under drought. The protective effects of HA were also evident for both PSI subpopulations. The enhanced tolerance was associated with the activation of antioxidant enzymes (CAT, SOD, APX) and the increased levels of anthocyanins and total phenolic content (TPC). In contrast, lower HA concentrations (1 and 3 mg/mL) provided insufficient protection. This study clearly demonstrates that HA enhances drought tolerance in basil in a concentration-dependent manner by protecting the structural and functional integrity of the photosynthetic apparatus, supporting its potential use as a foliar treatment to improve crop resilience under water-limited conditions.

## 1. Introduction

One of the main abiotic stresses limiting plant growth, productivity, and global food security is drought [1]. Water deficit significantly affects plants by impairing cellular metabolism and physiology, reducing membrane stability, and inhibiting plant growth and yield [2,3]. Photosynthesis is among the primary physiological processes disrupted by drought stress [4,5]. Drought-induced physiological, biochemical, and molecular alterations strongly impair photosynthetic efficiency [6,7]. Chloroplasts are known to be the primary sites of oxidative stress due to the light reactions of photosynthesis and, in particular, the imbalance between light capture and its utilization [8]. The increased levels of reactive oxygen species (ROS) lead to the disassembly of pigment–protein complexes, destabilization of thylakoid membranes, and inhibition of both photosystem I (PSI) and photosystem II (PSII) activities [9,10]. Drought-induced dehydration causes significant disorder in the photosynthetic machinery, as evidenced by the loss of several PSI and PSII reaction center proteins and associated light-harvesting complexes under severe drought stress [11]. The authors demonstrated a decrease in PSII dimers, along with an increase in LHCII monomers in the LHCII-PSII supercomplexes. These changes disrupt the balance of electron-transport pathways and reduce photochemical efficiency [7,11,12,13,14]. PSII is known to be more sensitive than PSI under environmental stress [15,16]. Studies have shown that, under moderate drought, the core protein content of PSI often remains unchanged [17], whereas prolonged drought can reduce the size of the PSI antenna system and lead to the disassembly of the PSI supercomplexes, thereby disrupting the structure required for efficient energy transfer [9].

Plants have developed adaptive mechanisms such as the activation of antioxidant defenses and osmotic adjustments to alleviate the negative impacts of environmental stressors [1,6,18]. One promising approach for improving plant resilience and tolerance to abiotic stresses, including drought, is the use of plant biostimulants [19,20,21]. Biostimulants represent a diverse group of substances—such as humic acids, seaweed extracts, protein hydrolysates, and microbial inoculants—that can modulate plant physiological and metabolic processes, enhancing stress tolerance and crop performance [22,23]. Natural organic substances such as humic acid (HA) have attracted considerable attention due to their multifaceted roles in promoting plant growth [24], nutrient uptake [25], and stress adaptation [26]. HA has been shown to enhance the activity of antioxidant enzymes (such as superoxide dismutase, catalase, and peroxidases), thereby reducing ROS accumulation, mitigating oxidative damage, and protecting the photosynthetic apparatus [27,28,29,30]. Activation of antioxidant enzyme systems under drought stress following HA application has been demonstrated in different plant species, including maize, sorghum, and lettuce [28,30,31]. Foliar-applied HA (0.2 mg/mL) under drought increased total chlorophyll content and overall photosynthetic activity in wheat varieties [32]. Increased photosynthetic pigment content and improved relative water content (RWC) were also observed in spearmint (*Mentha spicata* L.) after the foliar application of 1 mg/mL HA [33]. HA has been shown to influence the expression of the stress-related antioxidant enzyme genes and effectively reduce drought stress [34]. The study of Alsamadany [35] has revealed that the HA application upregulated the expression of drought-related genes. However, the alleviating effects of HA on drought-induced changes vary among plant species [30]. Additionally, HA promotes osmotic adjustment by increasing the accumulation of compatible solutes, which stabilize membrane structures under water deficit [26,29]. The effectiveness of HA is known to be highly dose-dependent, with sub-optimal or supra-optimal doses producing insignificant or even inhibitory effects [36,37]. Despite many studies on the effects of HA on plants, the exact protective mechanisms underlying the primary processes of photosynthesis under drought conditions have not been elucidated.

Basil (*Ocimum basilicum* L. Italiano classico) is a widely used herb valued both as a fresh vegetable and in the pharmaceutical industry due to its essential oil content and secondary metabolites such as phenolic compounds, flavonoids, and anthocyanins [38,39]. Drought stress limits basil production worldwide, reducing basil biomass and altering metabolic composition, thereby compromising its economic and medicinal value [40,41,42]. Therefore, exploring effective approaches to enhance basil tolerance to drought is essential for maintaining productivity and quality under water-limited conditions.

The present study aimed to assess the effects of foliar-applied HA (1, 3, and 5 mg/mL) on the function of the photosynthetic apparatus under PEG-induced drought stress and to identify the most effective HA concentration within the tested range for protecting sweet basil (*Ocimum basilicum* L. Italiano classico) under drought conditions. In addition, the relationship between changes in the primary processes of photosynthesis and alterations in pigment composition, membrane stability, RWC, and antioxidant activity was examined. The results provide new insights into the role of HA in enhancing basil tolerance to drought stress and demonstrate that 5 mg/mL HA is the most effective concentration within the tested range.

## 2. Results

### 2.1. Pigment Composition

The effects of HA on pigment content in basil plants under PEG-induced drought stress varied depending on its concentration (Table 1). The data revealed that treatment with 20% PEG alone significantly reduced the amounts of chlorophyll *a* (Chl *a*) and carotenoids (Car) by 20% and 16%, respectively, while the amount of chlorophyll *b* (Chl *b*) remained unchanged (Table 1). Under PEG-induced drought stress, the foliar application of a higher HA concentration (5 mg/mL) increased the amounts of Chl *a* and Car by 41% and 45%, respectively, compared to plants treated with PEG alone. In contrast, the combined treatment with PEG and lower HA concentrations (1 and 3 mg/mL) did not alter Chl *a* and Car content, as their amounts did not differ significantly from those in PEG-treated plants (Table 1).

### 2.2. Membrane Stability Index and Relative Water Content

**Figure 1:**
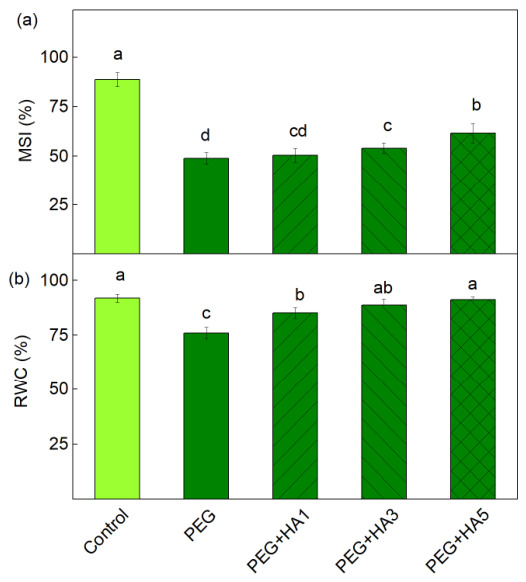
Influence of HA (1, 3 and 5 mg/mL) on (**a**) the membrane stability index (MSI) and (**b**) relative water content (RWC) of basil leaves under PEG-induced drought stress. The variants are labeled as follows: control; PEG treatment without HA (PEG); PEG treatment with 1 mg/mL HA (PEG+HA1); PEG treatment with 3 mg/mL HA (PEG+HA3) and PEG treatment with 5 mg/mL HA (PEG+HA5). The different letters show significant differences between the variants for the respective parameter at *p* < 0.05.

Membrane stability index (MSI) and relative water content (RWC) were used to determine the role of HA in basil tolerance under PEG-induced drought stress. The MSI decreased by 45% after PEG treatment compared to the control. The foliar application of 3 mg/mL HA under drought stress increased MSI by 10% relative to PEG treatment alone, whereas no significant difference was observed at 1 mg/mL HA. Application of 5 mg/mL HA under PEG-induced stress increased MSI by 26%, although the value remained lower than that of the control plants (Figure 1a). The RWC also decreased after treatment with PEG alone, while after applying all concentrations of HA, the values of this parameter increased. After applying 5 mg/mL HA under stress, the RWC level was similar to that of the control plants (Figure 1b).

### 2.3. Oxidative Stress Markers

**Figure 2:**
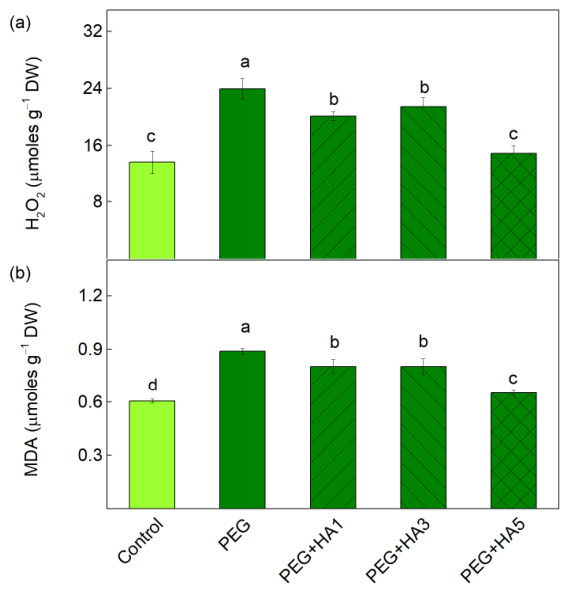
Influence of HA (1, 3 and 5 mg/mL) on the content of H_2_O_2_ (**a**) and MDA (**b**) in basil leaves under PEG-induced drought stress. The variants are labeled as follows: control; PEG treatment without HA (PEG); PEG treatment with 1 mg/mL HA (PEG+HA1); PEG treatment with 3 mg/mL HA (PEG+HA3) and PEG treatment with 5 mg/mL HA (PEG+HA5). Different letters show significant differences between the studied variants for the respective parameter at *p* < 0.05.

PEG-induced drought stress led to a 75% increase in H_2_O_2_ levels compared to the control plants (Figure 2a). The foliar application of HA under PEG treatment reduced these H_2_O_2_ levels relative to plants exposed to PEG alone. The decreases ranged from 11% to 16% at the lower HA concentrations (1 and 3 mg/mL). The lowest level of H_2_O_2_ (a reduction of 38%) was observed at 5 mg/mL HA, and the amount of H_2_O_2_ was similar to that of the control plants (Figure 2a). The amount of MDA, an indicator of lipid peroxidation, increased by 47% after PEG treatment (Figure 2b). All studied HA concentrations reduced MDA relative to PEG treatment, with the strongest reduction observed at the highest HA concentration (5 mg/mL); however, the control values were not reached (Figure 2b).

### 2.4. Antioxidant Enzymes, Anthocyanins and Total Phenolic Content

**Figure 3:**
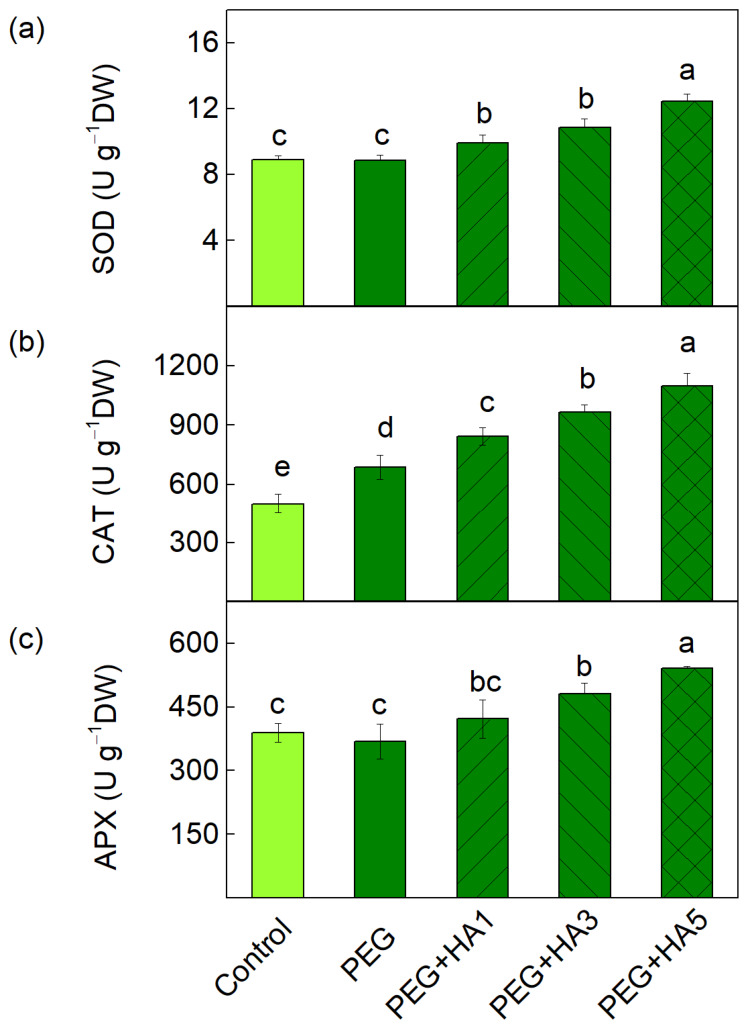
Activities of antioxidant enzymes: (**a**) superoxide dismutase (SOD), (**b**) catalase (CAT) and (**c**) ascorbate peroxidase (APX) in basil leaves under PEG-induced stress and HA application. The variants are labeled as follows: control; PEG treatment without HA (PEG); PEG treatment with 1 mg/mL HA (PEG+HA1); PEG treatment with 3 mg/mL HA (PEG+HA3) and PEG treatment with 5 mg/mL HA (PEG+HA5). Data are presented as mean values (±SE). Different letters indicate significant differences between variants (*p* < 0.05) for the respective enzymes.

The alterations in the enzyme activities of superoxide dismutase (SOD), catalase (CAT) and ascorbate peroxidase (APX) after the foliar application of HA under PEG-induced drought stress are presented in Figure 3. Treatment with PEG alone increased CAT activity by 37% compared to the control plants, while SOD and APX activities remained unchanged. The results demonstrated that the foliar application of HA under PEG-induced drought stress leads to the increased activities of all three antioxidant enzymes (SOD, CAT, and APX) compared to PEG-treated plants. The degree of enzyme activation depended on the HA concentration, with the highest concentration (5 mg/mL) producing the strongest response. At this concentration, CAT activity showed the greatest increase (by 60%), while SOD and APX activities were increased by 40–45% compared to PEG-treated plants (Figure 3).

**Figure 4:**
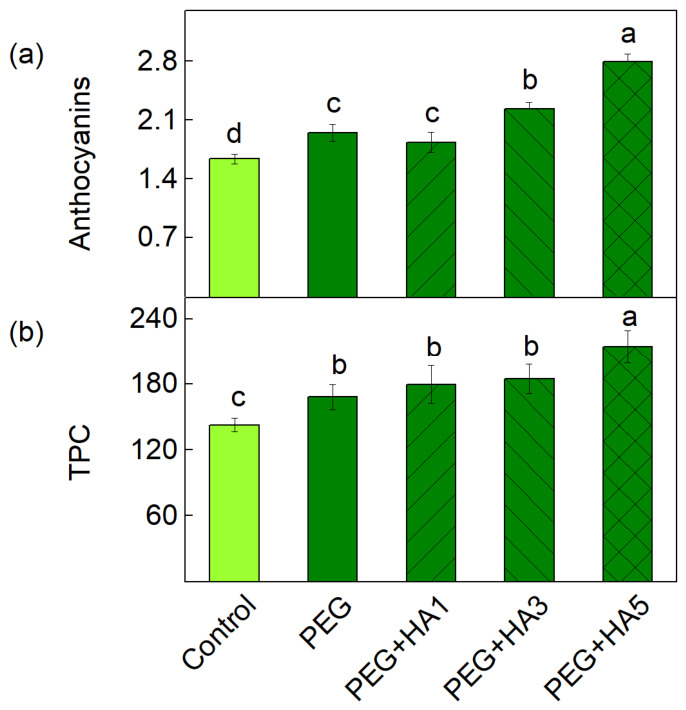
Influence of different HA concentrations (1, 3 and 5 mg/mL) on the content of anthocyanins (mg g^−1^ DW) (**a**) and TPC (mg g^−1^ DW) (**b**) in basil leaves under PEG-induced drought stress. The variants are labeled as follows: control; PEG treatment without HA (PEG); PEG treatment with 1 mg/mL HA (PEG+HA1); PEG treatment with 3 mg/mL HA (PEG+HA3) and PEG treatment with 5 mg/mL HA (PEG+HA5). Data are presented as mean values (±SE). Different letters indicate significant differences between variants (*p* < 0.05) for the respective parameters.

PEG-induced drought stress increased the anthocyanin amount and total phenolic content (TPC) in basil leaves (Figure 4). Anthocyanins increased by 19% compared to untreated plants (Figure 4a), and foliar spraying with 1 mg/mL of HA did not induce alterations in their level compared to PEG treatment alone. At the same time, HA spraying with 3 and 5 mg/mL enhanced anthocyanin accumulation by 36% and by 70%, respectively, compared to the control (Figure 4a). Similarly, PEG treatment alone increased TPC by 18% relative to the control plants. The application of lower HA concentrations (1 and 3 mg/mL) did not affect TPC compared to PEG treatment, while a significant increase in TPC (50%) was observed only after treatment with 5 mg/mL HA under stress compared to untreated plants (Figure 4b).

### 2.5. Pulse Amplitude-Modulated Chlorophyll a Fluorescence

**Figure 5:**
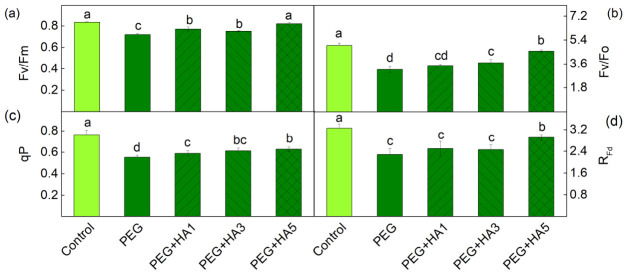
Influence of different HA concentrations (1, 3 and 5 mg/mL) under PEG-induced drought stress on the selected PAM parameters: (**a**) the maximum quantum yield of PSII (Fv/Fm); (**b**) the ratio of the photochemical to non-photochemical processes (Fv/Fo); (**c**) the coefficient of photochemical quenching (qP) and (**d**) the chlorophyll fluorescence decay ratio R_Fd_. The variants are labeled as follows: control; PEG treatment without HA (PEG); PEG treatment with 1 mg/mL HA (PEG+HA1); PEG treatment with 3 mg/mL HA (PEG+HA3) and PEG treatment with 5 mg/mL HA (PEG+HA5). Different letters indicate significant differences between variants (*p* < 0.05) for the respective parameter.

Pulse Amplitude-Modulated (PAM) chlorophyll *a* fluorescence was used to assess the impact of HA under PEG-induced drought stress on the functions of the photosynthetic apparatus of basil plants. PEG treatment led to a reduction in the maximum quantum yield of PSII (Fv/Fm) and in the ratio of photochemical to non-photochemical processes (Fv/Fo) as Fv/Fm decreased by 14% and Fv/Fo by 37% (Figure 5). In addition, reductions in the photochemical quenching (qP) and photosynthetic rate (R_Fd_) were also observed. The application of HA under drought stress increased Fv/Fm (by 7–39%) and Fv/Fo (16–44%), with the strongest effects observed at 5 mg/mL HA (Figure 5). After applying 5 mg/mL HA, a significant increase was also registered in R_Fd_ values (Figure 5).

**Figure 6:**
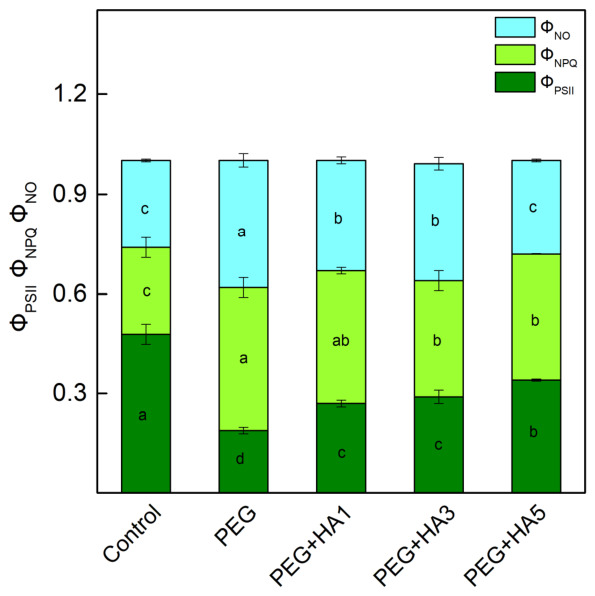
Influence of different HA concentrations (1, 3 and 5 mg/mL) under PEG-induced drought stress on the effective quantum yield of photochemical energy conversion of PSII (Φ_PSII_), the non-regulated energy losses (Φ_NO_) and the regulated energy losses (Φ_NPQ_). The variants are labeled as follows: control; PEG treatment without HA (PEG); PEG treatment with 1 mg/mL HA (PEG+HA1); PEG treatment with 3 mg/mL HA (PEG+HA3) and PEG treatment with 5 mg/mL HA (PEG+HA5). Different letters indicate significant differences between variants (*p* < 0.05) for the respective parameters.

PEG-induced drought stress decreased the effective quantum yield of PSII photochemistry (Φ_PSII_) by 60% and increased both non-regulated (Φ_NO_) and regulated (Φ_NPQ_) energy dissipation by 46% and 65%, respectively, compared to control plants (Figure 6). The application of HA under stress conditions prevented the photochemical activity of PSII (Φ_PSII_), as the effect was more pronounced at 5 mg/mL HA. At this concentration of HA, Φ_NO_ and Φ_NPQ_ decreased by 26% and 12%, respectively, relative to PEG-treated plants (Figure 6).

**Figure 7:**
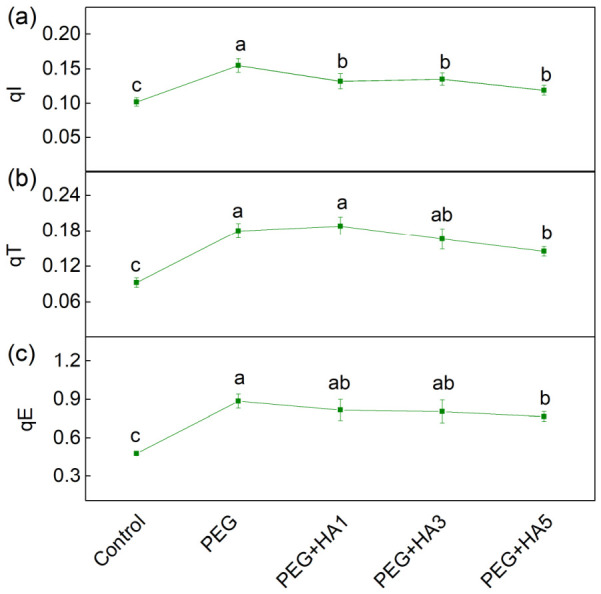
Influence of different HA concentrations (1, 3 and 5 mg/mL) under PEG-induced drought stress on the components of the non-photochemical quenching in green basil: (**a**) photoinhibitory quenching (qI), (**b**) state-transition quenching (qT), and (**c**) energy-dependent quenching (qE). The variants are labeled as follows: control; PEG treatment without HA (PEG); PEG treatment with 1 mg/mL HA (PEG+HA1); PEG treatment with 3 mg/mL HA (PEG+HA3), and PEG treatment with 5 mg/mL HA (PEG+HA5). Different letters indicate significant differences between variants (*p* < 0.05) for the respective parameters.

More detailed information on non-photochemical quenching within the photoprotective machinery is provided by its components: qI (photoinhibitory quenching), qT (state-transition quenching), and qE (energy-dependent quenching) (Figure 7). In response to PEG treatment, all these components increased compared to the control (Figure 7). Treatment with PEG increased qT, qE and qI by 96%, 85% and 52%, respectively (Figure 7). The application of HA under stress conditions reduced qI by 13–24% compared to PEG-treated plants. Compared to PEG treatment alone, the components qE and qT showed significant decreases by 14% and 20%, respectively, only at the highest HA concentration (5 mg/mL) (Figure 7).

The chlorophyll fluorescence after a single saturating pulse was deconvoluted into two components, fast (A_1_) and slow (A_2_), with times *t*_1_ and *t*_2_, respectively. These components characterize the distinct electron pathways of Q_A_ reoxidation [43,44]. PEG treatment alone led to an increase in *t*_1_ of 31% and a decrease in *t*_2_ of 13% compared to control plants. The application of lower HA concentrations (1 and 3 mg/mL) under drought conditions had a smaller effect on the time *t*_1_ compared to PEG-treated plants, whereas 5 mg/mL HA decreased this time to the level of the control plants. The time *t*_2_ increased at all studied concentrations of HA compared to the plants treated with PEG alone. The ratio of two components (A_1_/A_2_) decreased under PEG treatment alone, but only after the application of the highest HA concentration (5 mg/mL), this ratio increased (Table 2).

### 2.6. Fast Chlorophyll a Fluorescence

**Figure 8:**
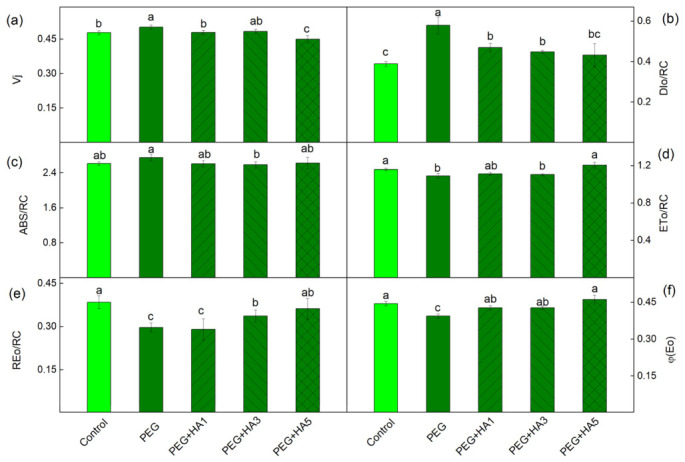
Selected JIP parameters of basil plants under PEG treatment and HA applied at concentrations of 1, 3, and 5 mg/mL: (**a**) relative variable fluorescence at the J step, indicating the reduction state of Q_A_ (Vj); (**b**) dissipated energy flux per reaction center (DIo/RC); (**c**) absorption flux per reaction center (ABS/RC); (**d**) electron transport flux from Q_A_ to Q_B_ per PSII (ETo/RC); (**e**) electron flux reducing end acceptors at the PSI acceptor side (REo/RC); and (**f**) quantum yield of electron transport (φEo). The variants are labeled as follows: control; PEG treatment without HA (PEG); PEG treatment with 1 mg/mL HA (PEG+HA1); PEG treatment with 3 mg/mL HA (PEG+HA3) and PEG treatment with 5 mg/mL HA (PEG+HA5). Mean values (±SE) were calculated from 20 independent measurements. All parameters are expressed in relative units. Different letters indicate significant differences between variants for the respective parameters at *p* < 0.05.

**Figure 9:**
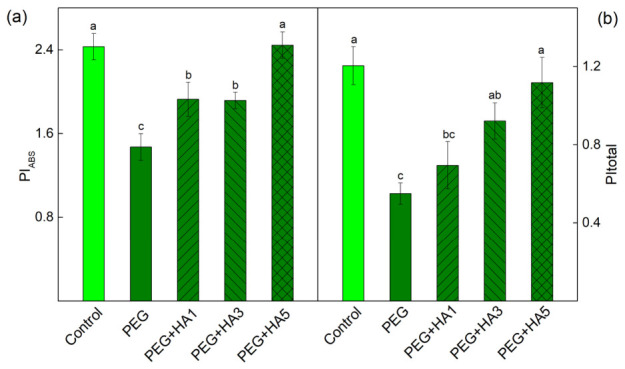
Performance indices of basil plants under PEG-induced drought stress and HA applied at concentrations of 1, 3, and 5 mg/mL: (**a**) performance index on an absorption basis (PI_ABS_) and (**b**) total performance index (PItotal). The variants are labeled as follows: control; PEG treatment without HA (PEG); PEG treatment with 1 mg/mL HA (PEG+HA1); PEG treatment with 3 mg/mL HA (PEG+HA3) and PEG treatment with 5 mg/mL HA (PEG+HA5). Mean values (±SE) were calculated from 5 independent measurements (n = 20). All parameters are expressed in relative units. Different letters indicate significant differences between variants for the respective parameters at *p* < 0.05.

More detailed information about the impact of HA under PEG-induced drought stress is provided by chlorophyll fluorescence induction (OJIP transitions). The experimental results showed that the antenna size per active reaction center (ABS/RC), the dissipation energy flux per reaction center (DIo/RC), and the instability of the PSII acceptor side (Vj) increased after PEG treatment. Among all examined parameters, the strongest PEG-induced increase was observed for DIo/RC (by 50%), whereas the most pronounced decrease occurred in REo/RC (by 24%), highlighting that PEG-induced stress markedly enhances energy dissipation while severely limiting electron flow toward PSI acceptors (Figure 8). A decrease was also registered for PI_ABS_ (by 38%) and PItotal (by 55%) (Figure 9). The application of HA prevented the PEG-induced drought changes in the photosynthetic apparatus (Figure 8). The protective effect of HA was most pronounced at 5 mg/mL, where the values of the studied parameters were similar to those of the control plants (Figure 8 and Figure 9).

The increased values of both performance indices after combined HA and PEG treatment resulted from the better protection of the performance of the primary photochemistry [φ(Po)/(1 − φ(Po))] and the non-light-dependent reaction [ψ(Eo)/(1 − ψ(Eo))] (Table S1). In addition, the higher values of PItotal after applying 5 mg/mL HA were determined by the better efficiency of electron transfer from Q_B_ to PSI electron acceptors [δ(Ro)/(1 – δ(Ro))] (Table S1).

### 2.7. P700 Photooxidation

The photooxidation of P700 (P700^+^) under far-red (FR) light was analyzed to evaluate PSI photochemistry (Table 3). The dark-decay kinetics of P700^+^ were deconvoluted into two exponential components: fast A_1_ with time t_1_^P700^ and slow A_2_ with time t_2_^P700^. The PEG treatment decreased relative changes in P700^+^ (ΔA/A) by 19%, time t_1_^P700^ by 27% and the ratio of amplitudes of the fast to slow components (A_1_^P700^/A_2_^P700^) by 50% (Table 3). The application of HA led to an increase in the ΔA/A ratio by 15–16% compared to PEG-treated plants, but values are smaller than those of the control plants. Data also revealed that t_2_^P700^ increased by 21% under PEG treatment, whereas it decreased after foliar treatment with all studied concentrations of HA. Treatment with 5 mg/mL HA under stress increased the A_1_^P700^/A_2_^P700^ ratio, reaching control values (Table 3).

## 3. Discussion

Drought stress is known to negatively impact plants by impairing photosynthesis, which is essential for their growth and productivity [1,7]. Although several studies have highlighted the important role of HA in regulating photosynthesis and enhancing plant tolerance to abiotic stress, detailed information regarding their influence on the photosynthetic apparatus remains insufficient [26,45,46]. Therefore, this study aimed to provide more detailed information about the protective effects of HA on the primary processes of photosynthesis in basil plants by assessing its influence on the function of the main complexes of the photosynthetic apparatus under PEG-induced drought stress. The physiological responses observed in plants following the foliar application of HA are likely mediated by a combination of nutrient uptake processes, surface interactions, and signaling-driven adjustments [47]. The humic substances are known to activate membrane-associated receptors and ion transport systems, triggering early signaling events such as transient ROS accumulation, Ca^2+^ fluxes, modulation of auxin- and ABA-related pathways and upregulation of the expression of drought-related genes [35,48,49].

PEG-induced drought stress causes a significant disruption of the photosynthetic machinery, accompanied by changes in leaf pigment content [11]. A reduction in photosynthetic pigments is a typical symptom of drought stress and is closely associated with alterations in plant morphology [14,50]. The data in this study revealed a decrease in the amounts of Chl *a* and Car, accompanied by an increase in H_2_O_2_ content in basil plants under stress conditions (Table 1, Figure 2), suggesting that the reduction in these pigments (Chl *a* and Car) is mainly due to oxidative stress. At the same time, no changes in the amount of Chl *b* were registered under water deficiency conditions (Table 1), which suggests a higher sensitivity of Chl *a* than Chl *b*. The decrease in Chl content is commonly observed in many plant species under drought stress, as the changes strongly depend on the plant sensitivity [14,51,52,53]. The Chl reduction may be due to stress-induced impairment in pigment biosynthetic pathways or pigment degradation [54,55].

Oxidative stress also caused increased lipid peroxidation, corresponding to an increased MDA content (Figure 2). All these changes, together with the decrease in RWC, led to reduced membrane stability, i.e., the MSI parameter decreased by 45% (Figure 1). The highest applied concentration of HA (5 mg/mL) prevented a decrease in RWC, Chl *a*, and Car, which in turn preserved membrane integrity (MSI). Smaller lipid peroxidation and membrane injury in drought-tolerant genotypes of *Brassica napus* and *Setaria italica* have been shown [56,57]. Having in mind that lipid peroxidation and membrane injury can be used to assess the plant drought tolerance [58,59], it could be suggested that 5 mg/mL HA increased basil tolerance under drought stress.

The protective effects of HA under PEG-induced drought stress were also associated with the activation of the antioxidant defense system. The current results revealed that, under stress conditions, HA application gradually activated SOD, CAT, and APX with the maximum effect observed at 5 mg/mL, whereas after PEG treatment alone, stimulation was registered only for CAT activity (Figure 3). The upregulation of these enzymes enhances the detoxification of superoxide radicals and hydrogen peroxide, thereby limiting oxidative damage to the photosynthetic membranes. Similar responses have been observed in previous studies, showing that HA improved antioxidant capacity in finger millet, wheat, foxtail millet, and maize [27,28,53,60,61,62]. It has been demonstrated that HA influences the transcription of stress-responsive antioxidant enzyme genes, contributing to a reduction in drought-induced damage [34]. HA treatment also promoted the accumulation of non-enzymatic antioxidants, including anthocyanins and TPC, which increased by 70% and 50%, respectively, at 5 mg/mL HA (Figure 4). These metabolites are known to contribute to ROS scavenging and support osmotic adjustment under stress conditions [26,29,63,64] as well as the functions of the photosynthetic apparatus [65]. The enhanced antioxidant capacity and osmotic stabilization of cellular structures after the foliar application of HA most likely contribute to the maintenance of membrane integrity and photosynthetic functions. Thus, the membrane integrity (MSI) was protected, and the RWC was restored to control levels after the application of 5 mg/mL HA, indicating that HA effectively mitigated the water deficit in leaf tissues. It has also been found that the modulation of the water status and antioxidant activity in wheat leaves are the reasons for the reduction in Cd toxicity in wheat leaves [66].

PEG-induced drought stress also led to a significant reduction in all studied PAM parameters, especially in the ratio of the photochemical to non-photochemical processes (Fv/Fo), the amount of the open reaction centers’ proportion (qP) and the photosynthetic rate (R_Fd_) (Figure 5). A similar decrease in photosynthetic efficiency and damage to PSII reaction centers under drought stress has been reported in barley, maize, chickpea, wheat and quinoa [15,53,67,68]. The strong decrease in Fv/Fo under drought stress (Figure 5) suggests structural alterations in the thylakoid membranes and an inhibition of the oxygen-evolving complex [54,69]. Analysis of the OJIP transition revealed an increase in Vj, which corresponds to altered Q_A_ reoxidation kinetics and restricted electron transfer from Q_A_^−^ to the plastoquinone pool (Figure 8, Table 2). These observations indicate changes in both the acceptor side (parameter Vj) and donor side (ratio Fv/Fo) of PSII. The drought-induced changes in the PSII complex could be the result of the alterations in D1 protein and Q_B_ reducing complex and the damage of the oxygen-evolving complex [70,71,72,73]. The drought-induced modification of PSII strongly limits the effective quantum yield of the photochemical energy conversion of PSII (Φ_PSII_) and the efficiency of the photosynthetic machinery and increases energy dissipation per reaction center (DIo/RC), which reflects structural and energetic constraints within PSII reaction centers (Figure 8). In addition, the increases in both regulated (Φ_NPQ_) and non-regulated (Φ_NO_) energy losses, revealing the real-time regulatory adjustments of energy dissipation under illumination, were registered (Figure 6). It is well known that non-photochemical quenching is a major photoprotective mechanism of the photosynthetic apparatus under stress [15]. Furthermore, our data revealed an increase in energy-dependent quenching component (qE, by 85%) and the state-transition quenching component (qT, by 96%) under PEG treatment (Figure 7). The strongly enhanced component qT indicates the stimulated/increased redistribution of excitation energy between the two photosystems, which is vital for protecting the photosynthetic apparatus under stress [74]. The ratio of these protective mechanisms (qE/qT) was 4.92 under stress compared to 5.19 in control plants, suggesting a decrease in the proportion of qE (i.e., ΔpH-dependent energy dissipation) in non-photochemical processes after PEG treatment alone. At the same time, photoinhibitory quenching (qI) increased, indicating PSII damage as a result of ROS accumulation [74,75]. The impairment of PSII function also influenced electron flow toward PSI acceptors (REo/RC) and reduced PItotal (Figure 8 and Figure 9).

PEG-induced drought stress also influenced PSI activity (ΔA/A) and P700 photooxidation kinetics, including changes in the fast and slow components of P700^+^ dark reduction (Table 3). Previous studies have shown that drought stress reduces PSI antenna complexes and affects the organization of PSI-LHCI supercomplexes [9]. This study showed that drought stress caused an increase in t_2_^P700^, as well as a decrease in t_1_^P700^ and the A_1_/A_2_ ratio under drought conditions (Table 3), suggesting changes in the two PSI populations associated with different thylakoid domains [76,77]. The decrease in t_1_^P700^ indicates an increased cyclic electron flow, a process that protects the photosynthetic machinery from oxidative damage [78].

The highest concentration of HA (5 mg/mL) provided the optimal protection of photosynthetic function under PEG-induced drought stress. Foliar HA alleviated the drought-induced changes on both the donor and acceptor sides of PSII. Analysis of the dark relaxation of chlorophyll *a* fluorescence showed that, after HA application under stress, the times of the fast and slow components (*t*_1_ and *t*_2_) were similar to those of the untreated plants (Table 2). The fast component (A_1_ and time *t*_1_) is associated with Q_A_ reoxidation by the PQ pool, while the slow component (A_2_ and time *t*_2_) is associated with Q_A_ reoxidation by recombination with S_2_ and S_3_ states of the oxygen-evolving complex [79,80,81]. This study demonstrated that HA preserves the functionality of the PSII acceptor side by maintaining efficient Q_A_ reoxidation and alleviating its interaction with plastoquinone, as evidenced by the decrease in *t*_1_ (Table 2).

Additionally, HA treatment increased the Fv/Fo ratio (Figure 5), indicating an improvement in the PSII donor side. All these changes in the PSII complex after the HA application under stress led to an increased number of open PSII reaction centers (qP) and an improved effective quantum yield of PSII photochemistry (Φ_PSII_) (Figure 5 and Figure 6). The data also revealed an improved electron transport from Q_A_^−^ to Q_B_ (ETo/RC), a higher quantum yield of electron transport beyond Q_A_^−^ (φEo), and an increased electron flux reaching PSI end acceptors (REo/RC). The protection of PSII photochemistry during drought in the presence of HA corresponds with an improved photosynthesis rate (R_Fd_) (Figure 5 and Figure 8). Similarly, it has been found that the HA application improved the net photosynthesis under water stress by increasing the gas exchange rate and electron transport flux in rapeseed plants, as the parameters ABS/RC and ETo/RC were increased, and DIo/RC decreased after the application of HA [82]. The protective effect of HA on the functions of the photosynthetic apparatus was clearly shown by the performance indices (PI_ABS_ and PI total), which were similar to those of control plants (Figure 9). Analysis of their components revealed that HA prevented the drought-induced decline in primary photochemistry [φ(Po)/(1 − φ(Po))], the non-light-dependent reaction [ψ(Eo)/(1 − ψ(Eo))], and the efficiency of the electron transfer from Q_B_ to PSI electron acceptors [δ(Ro)/(1 – δ(Ro))] (Table S1). The better protection of the function of the photosynthetic apparatus corresponded with the decreased energy dissipation per reaction center (DIo/RC) and the reduced components of the non-photochemical quenching (qE, qT and qI) (Figure 7 and Figure 8). In addition, combined PEG and HA treatment increased the ratio of energy-dependent quenching (qE) to state-transition quenching (qT) to 5.28, compared with 4.92 under PEG alone, indicating a greater contribution of qE to the protection of the photosynthetic apparatus. This study also showed the protective role of HA on PSI photochemistry (Table 3). The times (t_1_^P700^ and t_2_^P700^) of P700 dark relaxation after HA treatment under stress were similar to those of the control, suggesting the effective protection of the two PSI subpopulations.

## 4. Materials and Methods

### 4.1. Plant Growth Conditions and Treatments

Seeds from sweet basil (*Ocimum basilicum* L. Italiano Classico) were grown hydroponically in containers on a half-strength Hoagland’s solution containing 2.5 mM KNO_3_, 2.5 mM Ca(NO_3_)_2_, 1 mM MgSO_4_, 0.5 mM NH_4_NO_3_, 0.5 mM K_2_HPO_4_, 23 µM H_3_BO_3_, 4.5 µM MnCl_2_, 0.4 µM ZnSO_4_, 0.2 µM CuSO_4_, 0.25 µM Na_2_MoO_4_, and 20 µM Fe-EDTA (pH 6.0) as in [65]. Cultivation was carried out under controlled conditions at a light intensity of 150 µmol photons m^−2^·s^−1^, a 12 h photoperiod, and a 24/20 °C (day/night) temperature. The nutrient solution in containers was renewed every three days. Drought stress was induced on one-month-old plants by adding 20% polyethylene glycol 6000 (PEG 6000) to the nutrient solution for three days. The foliar spraying of plants with three different concentrations (1, 3 or 5 mg/mL) of HA (Thermo Fisher Scientific, Waltham, MA, USA) was performed 24 h prior the PEG treatment. HA was dissolved in water (slightly alkaline, pH 7.8). The control group of plants was sprayed with an equivalent volume of water. After 3 days of the PEG exposure, all measurements were conducted on fully expanded mature basil leaves.

### 4.2. Determination of the Pigment Composition

The content of chlorophyll *a* (Chl *a*), chlorophyll *b* (Chl *b*) and carotenoids (Car) was determined by incubating fresh leaf tissues in 80% (*v*/*v*) acetone overnight at 4 °C in the darkness following the protocol described in [83]. Subsequently, the leaf extracts were filtered through Miracloth (475855-1R, Millipore, Merck, Darmstadt, Germany) before measuring the concentrations of photosynthetic pigments spectrophotometrically, following the method of Lichtenthaler [84].

### 4.3. Relative Water Content and Membrane Stability Index

The relative water content (RWC) of basil leaves was evaluated as described in [85]. The RWC was calculated using the following equation:

RWC (%) = (FW − DW)/(TW − DW) × 100

where FW is the fresh weight (measured immediately after harvesting the leaves), TW is the turgid weight (of leaves immersed in distilled water until full hydration), and DW is the dry weight (measured after oven-drying the leaves at 80 °C for 24 h). This parameter reflects the water status of the leaf tissues under different experimental treatments.

The cell membrane stability in leaf tissues was evaluated according to the method described in [86]. Leaf tissue samples taken from the control and differently treated plants were placed in 30 mL of distilled water and left to incubate at room temperature for 24 h. After incubation, the initial electrical conductivity (EC1) of the solutions was measured using a conductivity meter (Hydromat LM302, Witten, Germany). The solutions were then subjected to 20 min of boiling to completely disrupt the membranes, after which they were cooled to room temperature and the final conductance (EC2) was recorded again to assess the total ion leakage. The membrane stability index (MSI) was estimated in percentages using the following equation:

MSI (%) = [(1 − EC1/EC2)] × 100.

### 4.4. Determination of MDA and H_2_O_2_ Content

Lipid peroxidation was determined by the malondialdehyde (MDA) content using the protocol of Stewart and Bewley [87]. Absorbance values were recorded spectrophotometrically at 532 nm and 600 nm. The MDA concentration was expressed as µmoles per g DW using an extinction coefficient of 155 mM^−1^ cm^−1^.

Hydrogen peroxide (H_2_O_2_) content was determined following the method described in [88]. Absorbance was measured spectrophotometrically at 390 nm. The H_2_O_2_ concentration was calculated using a standard curve and expressed as µmoles per g DW.

### 4.5. Determination of Antioxidant Enzyme Activities

The activity of superoxide dismutase (SOD, U g^−1^ DW) was determined according to the method described in [89], based on its ability to inhibit the photochemical reduction of nitroblue tetrazolium (NBT). The absorbance was recorded at 560 nm. One unit of SOD activity was defined as the amount of enzyme required for 50% inhibition of NBT reduction. Catalase (CAT, U g^−1^ DW) activity was assayed following the monitoring of the decrease in absorbance at 240 nm over 2 min after the initiation of the reaction. Ascorbate peroxidase (APX, U g^−1^ DW) activity was determined by measuring the H_2_O_2_-dependent oxidation rate of ascorbic acid (AsA) over 1 min, according to the method described in [90].

### 4.6. Determination of Anthocyanins and Total Phenolic Content

The anthocyanins were determined according to the method described in [91]. Anthocyanin concentrations were determined spectrophotometrically by measuring the absorbances at 536 nm and 600 nm and expressed as mg of cyanidin-3-glucoside equivalent per g DW using the molar extinction coefficient of 33,000 mM^−1^ cm^−1^.

The total phenolic content (TPC) was determined using 10% Folin–Ciocalteu’s reagent as described in [92]. The absorbance was recorded spectrophotometrically at 765 nm, and the TPC values were expressed as mg gallic acid (GAE) per g DW.

### 4.7. Pulse Amplitude-Modulated Chlorophyll a Fluorescence Measurements

Pulse Amplitude-Modulated (PAM) chlorophyll *a* fluorescence was measured with a fluorometer (Model 101/103, Walz GmbH, Effeltrich, Germany) following an established procedure [80]. Prior to measurements, leaves were dark-adapted for 20 min. The minimum fluorescence in the dark (Fo) was recorded using modulated measuring light (1.6 kHz, 0.02 µmol m^−2^ s^−1^). The maximal dark-adapted fluorescence (Fm) was obtained with a saturating pulse (3000 µmol m^−2^ s^−1^, 0.8 s). Actinic illumination (150 µmol m^−2^ s^−1^) was applied using a Schott KL 1500 light source (Schott Glaswerke, Mainz, Germany), and steady-state fluorescence (Fs) was recorded. During illumination, saturating pulses were applied every 60 s to determine maximal light-adapted fluorescence (Fm′). The several photosynthetic parameters were used to evaluate PSII photochemistry (Table 4).

Non-photochemical quenching (NPQ) was analyzed by separating its major components: energy-dependent quenching (qE), state-transition quenching (qT) and photoinhibitory quenching (qI) (Table 4). After illumination, plants were returned to darkness, and fluorescence relaxation kinetics were followed for 15 min using saturating pulses at 1, 2, 3, 4, 5, 10, and 15 min to obtain Fm2 and Fm15, corresponding to the fast (2 min) and slow (15 min) phases of NPQ relaxation. The NPQ components were quantified from the decline in NPQ during dark relaxation using the following expressions from [93,94]:

qE = (Fm^2^ − Fm’)/Fm’

qT = (Fm^15^ − Fm^2^)/Fm^2^

qI = (Fm − Fm^15^)/Fm^15^

The chlorophyll fluorescence decay ratio (R_Fd_) was calculated as R_Fd_ = F_d_/Fs, where F_d_ is the decline from Fm to Fs under continuous saturating light [95].

PSII photochemical efficiency was evaluated using selected fluorescence parameters shown in Table 4.

Fluorescence relaxation kinetics in dark-adapted leaves were evaluated following excitation with a saturating light pulse (3000 µmol photons m^−2^ s^−1^). The relaxation curves could be fitted by two components (fast and slow components). The times of fast (*t*_1_) and slow (*t*_2_) components and the ratio of their amplitudes (A_1_/A_2_) were determined. The fast component characterizes the reoxidation of Q_A_^−^ through the plastoquinone pool, whereas the slower phase is linked to the recombination reactions associated with the S_2_ and S_3_ states of the oxygen-evolving complex [80].

### 4.8. Chlorophyll a Fluorescence Induction

OJIP transients were recorded to evaluate the effect of treatment with HA (1, 3, or 5 mg/mL) on the function of the photosynthetic apparatus under PEG-induced stress. Measurements were performed using a Handy PEA+ fluorimeter (Hansatech Instruments, King’s Lynn, UK), and fluorescence induction curves were analyzed with the PEA Plus software (v.1.13). Before each measurement, leaves were dark-adapted for 15 min. A saturating light pulse of 3200 µmol photons m^−2^ s^−1^ was then applied to induce the OJIP fluorescence rise. All measurements were taken from mature basil leaves. The OJIP transients were processed to obtain a comprehensive set of JIP-test parameters, summarized in Table 4.

### 4.9. P700 Photooxidation Measurements

Photooxidation of P700 (P700^+^) in leaves was assessed using a dual-wavelength detection system operating at 820 nm (ED 700DW-E, Walz, Effeltrich, Germany) coupled to a PAM-101E control unit in the reflectance mode, following the protocol as in [96]. The leaves were dark-adapted for 20 min before measurements. P700 oxidation was induced by far-red (FR) light provided by a 102-FR emitter (Walz GmbH, Effeltrich, Germany). Changes in P700 redox state were monitored as absorbance variations at 820 nm (ΔA) during FR exposure. From these measurements, the relative absorbance change (ΔA/A) was calculated, and the kinetics of P700^+^ reduction in darkness were analyzed. The decay of signal was deconvoluted into two exponential components characterized by fast and slow times (t_1_^P700^ and t_2_^P700^) along with respective amplitudes (A_1_^P700^ and A_2_^P700^) as described previously in [97].

**Table 4:** Chlorophyll *a* fluorescence parameters (PAM and JIP test) defined according to the methodologies described in [81,93,98,99,100,101].

Functional Group	Parameter and Description
	**PAM parameters**
Variable Fluorescence and Derived Ratios	Fv = Fm − Fo—variable fluorescence
	Fv/Fm—maximum quantum efficiency of PSII photochemistry in dark-adapted state
	Fv/Fo = (Fm − Fo)/Fo—balance between photochemical and non-photochemical processes
	Fv′ = Fm′ − Fo′—variable fluorescence in light-adapted state (if Fo′ is measured or estimated)
Quantum Yields of PSII	Φ_PSII_ = (Fm′ − Fs)/Fm′—effective quantum yield
	Φ_NO_ = Fs/Fm—non-regulated energy dissipation
	Φ_NPQ_ = (Fs/Fm′) − (Fs/Fm)—regulated energy dissipation via NPQ mechanisms.
Photochemical Quenching	qP = (Fm′ − Fs)/Fv′—photochemical quenching coefficient, fraction of open PSII reaction centers
Non-Photochemical Quenching Components	qE—energy-dependent quenching (ΔpH-dependent)
	qT—state-transition quenching
	qI—photoinhibitory quenching
Fluorescence-Based Physiological Indicators	R_Fd_ = Fd/Fs—fluorescence decay ratio, proxy for photosynthetic performance and vitality
	**JIP parameters**
	Vj—relative variable fluorescence at the J-step
Energy fluxes per reaction center	ABS/RC = 1/φPo—absorbed energy flux
	DIo/RC = (1 − φPo)/φPo—energy dissipated as heat
	ETo/RC= ψ (Eo)/φPo—electron transport flux from Q_A_^−^ to Q_B_
	REo/RC= (ψ (Eo) *×* δ (Ro))/φPo—electron flux reaching PSI end acceptors
Quantum yield	φEo—quantum yield of electron transport beyond Q_A_^−^
Performance indices	PI_ABS_—based on absorption and PSII efficiency PI_ABS_ = γ(RC)/(1 − γ(RC)) *×* φPo/(1 − φPo) *×* ψ (Eo)/(1 − ψ(Eo))
	PItotal—total, including PSI contribution PItotal = PI_ABS_ × δ (Ro)/(1 − δ (Ro))
Structural/derived PI components	γ(RC)/(1 − γ(RC))—ratio of active reaction centers to total chlorophyll
	φPo/(1 − φPo)—maximum of primary photochemistry
	ψ(Eo)/(1 − ψ(Eo))—probability of electron transport beyond Q_A_^−^
	δ(Ro)/(1 − δ(Ro))—efficiency/probability with which an electron from the intersystem electron carriers is transferred to reduce end electron acceptors at the PSI acceptor side

### 4.10. Statistical Analysis

Data are expressed as mean ± standard error (SE), obtained from two independent experiments with four biological replicates per treatment (n = 8). The statistical effects of the PEG+HA treatments were assessed using analysis of variance (ANOVA). When significant differences were detected, Tukey’s post hoc test was applied for multiple comparisons among means. Prior to analysis, data were examined to confirm normal distribution and homogeneity of variances. Statistical significance was accepted at *p* < 0.05. All calculations were performed using OriginPro 9.0 (OriginLab Corporation, Northampton, MA, USA).

## 5. Conclusions

The present study contributes to the expansion and deepening of existing knowledge on HA-mediated drought mitigation by providing a detailed mechanistic analysis of the protective effects of HA on the functions of photosynthetic apparatus and the related defense processes playing a regulatory role in basil plants exposed to PEG-induced drought stress. Drought stress caused substantial damage to both PSII and PSI. The inhibition of PSII photochemistry resulted from alterations in its donor and acceptor sides. Drought-induced changes at the acceptor side influenced Q_A_ reoxidation, consistent with a restriction of the interaction between Q_A_ and plastoquinone, reduced electron transport (ETR, REo/RC), and produced pronounced declines in performance indices (PI_ABS_, PItotal). The decreased efficiency of the photosynthetic apparatus after PEG treatment was further associated with elevated oxidative stress (H_2_O_2_), enhanced lipid peroxidation, reduced RWC, and altered pigment composition, all of which correspond to a disruption of the membrane integrity. The data also highlight the influence of both PSI subpopulations under drought stress. The protective effect of foliar HA was strongly dose-dependent, with lower concentrations having negligible effects on basil plants. The foliar spraying of 5 mg/mL HA markedly protects the primary processes of photosynthesis by preventing PEG-induced disruptions in the PSII donor and acceptor sides. This protection corresponded with improved Q_A_ reoxidation by plastoquinone and enhanced electron transport efficiency toward the end PSI acceptors. Performance indices clearly reflected the mitigating role of HA on the photosynthetic apparatus under PEG-induced drought stress. At the same time, HA stimulated the activity of antioxidant enzymes (CAT, SOD, APX) and decreased oxidative stress markers (H_2_O_2_ and MDA). These findings indicate that HA-induced modulation of antioxidant enzymes and stabilization of the photosynthetic apparatus occurred simultaneously, with each process reinforcing the other under drought stress.

## Figures and Tables

**Table 1:** Influence of HA (1, 3 and 5 mg/mL) on chlorophyll *a* (Chl *a*), chlorophyll *b* (Chl *b*), and carotenoids (Car). The variants are labeled as follows: control; PEG treatment without HA (PEG); PEG treatment with 1 mg/mL HA (PEG+HA1); PEG treatment with 3 mg/mL HA (PEG+HA3) and PEG treatment with 5 mg/mL HA (PEG+HA5). Different letters show significant differences in the respective parameter at *p* < 0.05.

Variant	Chl *a* (mg/g DW)	Chl *b* (mg/g DW)	Car (mg/g DW)
Control	19.99 ± 1.01 ^a^	5.66 ± 0.28 ^a^	5.09 ± 0.25 ^b^
PEG	16.00 ± 1.33 ^b^	4.86 ± 0.25 ^a^	4.29 ± 0.17 ^c^
PEG+HA1	16.10 ± 2.68 ^b^	4.61 ± 0.42 ^a^	4.47 ± 0.76 ^bc^
PEG+HA3	16.56 ± 1.55 ^b^	5.33 ± 0.41 ^a^	4.52 ± 0.40 ^bc^
PEG+HA5	22.60 ± 2.22 ^a^	6.12 ± 0.57 ^a^	6.20 ± 0.52 ^a^

**Table 2:** Influence of HA (1, 3, and 5 mg/mL) on the dark relaxation of chlorophyll fluorescence induced by a single saturating light pulse in the leaves of green basil under PEG treatment. The following parameters are characterized: *t*_1_—the time constant of the fast component (A_1_); *t*_2_—the time constant of the slow component (A_2_); and A_1_/A_2_—the ratio of the amplitudes of the fast and slow components. The variants are labeled as follows: control; PEG treatment without HA (PEG); PEG treatment with 1 mg/mL HA (PEG+HA1); PEG treatment with 3 mg/mL HA (PEG+HA3) and PEG treatment with 5 mg/mL HA (PEG+HA5). Different letters indicate significant differences between variants for the respective parameters at *p* < 0.05.

Variant	*t*_1_ (s)	*t*_2_ (s)	A_1_/A_2_
Control	0.513 ± 0.037 ^b^	17.920 ± 0.688 ^a^	7.446 ± 0.293 ^a^
PEG	0.672 ± 0.041 ^a^	15.613 ± 0.076 ^b^	6.411 ± 0.221 ^c^
PEG+HA1	0.613 ± 0.027 ^ab^	17.537 ± 0.365 ^a^	6.261 ± 0.387 ^c^
PEG+HA3	0.613 ± 0.031 ^ab^	17.297 ± 0.708 ^a^	6.506 ± 0.302 ^bc^
PEG+HA5	0.561 ± 0.020 ^b^	17.876 ± 0.876 ^a^	7.128 ± 0.118 ^ab^

**Table 3:** Influence of HA (1, 3 and 5 mg/mL) on the relative changes in P700^+^ (ΔA/A), the fast and slow time constants (t_1_^P700^ and t_2_^P700^) and the ratio of the amplitudes of the fast and slow components (A_1_^P700^/A_2_^P700^) of P700^+^ dark reduction in basil under PEG-induced drought stress. The variants are labeled as follows: control; PEG treatment without HA (PEG); PEG treatment with 1 mg/mL HA (PEG+HA1); PEG treatment with 3 mg/mL HA (PEG+HA3) and PEG treatment with 5 mg/mL HA (PEG+HA5). The different letters show significant differences between variants in the respective parameter at *p* < 0.05.

Variant	t_1_^P700^ (s)	t_2_^P700^ (s)	A_1_^P700^/A_2_^P700^	ΔA/A
Control	2.778 ± 0.386 ^a^	34.483 ± 1.189 ^b^	3.03 ± 0.48 ^a^	9.79 ± 0.48 ^a^
PEG	2.041 ± 0.250 ^b^	41.667 ± 3.472 ^a^	1.52 ± 0.22 ^b^	7.93 ± 0.40 ^c^
PEG+HA1	2.941 ± 0.346 ^a^	27.778 ± 2.315 ^c^	2.16 ± 0.31 ^b^	9.14 ± 0.27 ^b^
PEG+HA3	2.941 ± 0.173 ^a^	35.714 ± 1.276 ^b^	2.02 ± 0.06 ^b^	9.22 ± 0.30 ^b^
PEG+HA5	2.439 ± 0.119 ^a^	35.714 ± 1.276 ^b^	2.53 ± 0.21 ^a^	9.10 ± 0.27 ^b^

## Data Availability

The original contributions presented in this study are included in the article/Supplementary Material. Further inquiries can be directed to the corresponding author.

## Supplementary Materials

The following supporting information can be downloaded at: https://www.mdpi.com/article/10.3390/plants15101491/s1, Table S1. Influence of different HA concentrations (1, 3 and 5 mg/mL) under PEG-induced drought stress on the components of the performance indices PI_ABS_ and PItotal.

## Abbreviations

APX	Ascorbate peroxidase
Car	Carotenoids
CAT	Catalase
Chl *a*	Chlorophyll *a*
Chl *b*	Chlorophyll *b*
HA	Humic acid
H_2_O_2_	Hydrogen peroxide
LHC	Light-harvesting complex
MDA	Malondialdehyde
MSI	Membrane stability index
PEG	Polyethylene glycol
PSI	Photosystem I
PSII	Photosystem II
ROS	Reactive oxygen species
RWC	Relative water content
SOD	Superoxide dismutase
TPC	Total phenolic content
