# Supplementary material for: Humic Acid Modulates Photosynthetic Responses to PEG-Induced Drought in Ocimum basilicum L
Source: Plants (Basel). 2026 May 13;15(10):1491. doi: 10.3390/plants15101491 (PMC13210831; doi:10.3390/plants15101491)
Supplement: Supplementary file 1 [file plants-15-01491-s001.zip › plants-4277448-supplementary.pdf]

Humic Acid Modulates Photosynthetic Responses to PEG-Induced Drought in *Ocimum basilicum* L.

Martin A. Stefanov, Georgi D. Rashkov, Preslava B. Borisova, Anelia G. Dobrikova and Emilia L. Apostolova*

Table S1. Influence of different HA concentrations (1, 3 and 5 mg/ml) under PEG-induced drought stress on the components of the performance indices PIABS and PItotal. The variants are labeled as follows: Control; PEG treatment without HA (PEG); PEG treatment with 1 mg/ml HA (PEG+HA1); PEG treatment with 3 mg/ml HA (PEG+HA3) and PEG treatment with 5 mg/ml HA (PEG+HA5). All parameters are expressed in relative units. Different letters indicate significant differences between variants for respective parameters at $p < 0.05$.

Variants	γ(RC)/(1-γ(RC))	φ(Po)/(1-φ(Po))	ψ(Eo)/(1-ψ(Eo))	δ(Ro)/(1-δ(Ro))
Control	0.383 ± 0.006[ab]	5.746 ± 0.108[a]	1.096 ± 0.039[ab]	0.506 ± 0.048[a]
PEG	0.371 ± 0.010[b]	4.189 ± 0.248[c]	0.977 ± 0.035[c]	0.364 ± 0.032[b]
PEG + HA1	0.384 ± 0.011[ab]	4.577 ± 0.125[b]	1.089 ± 0.043[ab]	0.359 ± 0.059[b]
PEG + HA3	0.415 ± 0.012[a]	4.398 ± 0.214[b]	1.057 ± 0.058[b]	0.51 ± 0.057[ab]
PEG + HA5	0.383 ± 0.032[ab]	5.129 ± 0.318[ab]	1.226 ± 0.078[a]	0.489 ± 0.089[a]
